# A multimodal context-aware AI recommender for smart farming

**DOI:** 10.1371/journal.pone.0359596

**Published:** 2026-10-08

**Authors:** John Telesphory Mhagama, Kanwal Garg

**Affiliations:** 1 Department of Computer Science and Applications, Kurukshetra University, Kurukshetra, Haryana, India; 2 Department of Research and Innovation, Mtech Mavens Company Limited, Dar es Salaam, Tanzania; Firat Universitesi, TÜRKIYE

## Abstract

Agricultural productivity in developing regions is significantly affected by plant diseases and pest infestations, making early detection and timely intervention essential for improving crop yield and food security. This study proposes a multimodal context-aware AI recommendation framework that integrates maize leaf image analysis and environmental conditions to support intelligent agricultural decision-making. The framework combines a deep learning-based image classification model with weather variables, including temperature, humidity, rainfall, and solar radiation, through a multimodal fusion model for maize disease diagnosis. Based on the predicted crop condition, a context-aware recommendation engine integrates rule-based agronomic knowledge with the Qwen2.5:7B large language model to generate reliable and actionable natural-language recommendations for farmers. The generated recommendations are subsequently evaluated using Llama 3.1:8B in a blind LLM-based evaluation framework. Experiments were conducted using maize leaf images from the YEESI Lab dataset and a 61-day environmental dataset from the NASA POWER database, obtained for the geographic coordinates of Morogoro, Tanzania. Three maize conditions were considered: healthy plants, aphid infestation, and maize streak virus infection. The image-based model achieved an accuracy of 91%, while the weather-based model showed lower performance due to overlapping environmental characteristics among disease classes. The proposed multimodal fusion model achieved a classification accuracy of 94%, demonstrating the effectiveness of integrating visual and environmental information for disease diagnosis. The recommendation engine achieved an overall evaluation score of 4.80/5.00 across five quality dimensions—Safety, Technical Accuracy, Relevance, Actionability, and Clarity—indicating that the generated recommendations were agronomically consistent, context-aware, and actionable. The proposed framework provides an end-to-end AI-driven decision-support solution that integrates multimodal disease diagnosis, and context-aware recommendation for precision agriculture.

## Introduction

Agriculture remains a primary source of livelihood in many developing countries, particularly in Sub-Saharan Africa [1,2]. However, crop diseases and pest infestations significantly lessen agricultural productivity and threaten global food security [3]. According to the Food and Agriculture Organization (FAO), up to 40% of global crop production is lost annually due to plant pests and diseases [4]. Among staple crops, maize plays a critical role in ensuring nutritional and economic stability for rural communities. Globally, maize losses caused by insect pests and pathogens are estimated at 22.5% of total production [5] Diseases such as maize streak virus and pests such as aphids can cause substantial yield losses if not detected and controlled early [6,7]. Traditional crop disease diagnosis and actionable recommendations rely heavily on manual inspection by farmers or agricultural experts [8]. This process is often time-consuming, subjective, and limited by the availability of expert knowledge in rural areas. Advances in artificial intelligence and machine learning have introduced new opportunities for automated crop monitoring systems that can assist farmers in identifying diseases and endorsing appropriate interventions [6,8,9].

Recent studies have demonstrated the effectiveness of deep learning techniques in plant disease detection using leaf images [9–11]. Convolutional neural networks (CNNs) can automatically learn visual patterns associated with plant health conditions [6,12]. However, relying solely on visual information may overlook important contextual factors such as environmental conditions, which often influence disease development and pest activity. Environmental variables such as temperature, humidity, rainfall, and solar radiation play an important role in crop growth and disease propagation [13,14]. Recent advances in multimodal artificial intelligence have demonstrated that integrating environmental information with image-based diagnosis improves predictive robustness by combining complementary sources of information [15–17]. Furthermore, emerging research has explored explainable artificial intelligence and large language models to enhance the interpretability and usability of agricultural AI systems [18,19].

Despite these advances, two important research challenges remain. First, many state-of-the-art models are developed and evaluated using curated benchmark datasets, resulting in high laboratory accuracy but reduced generalization under real-world field conditions characterized by varying illumination, backgrounds, and environmental variability [20]. Second, existing studies primarily emphasize disease detection and classification, with comparatively limited attention given to integrating prediction models with intelligent recommendation mechanisms that transform disease predictions into practical, context-aware agronomic advice for farmers [19]. These limitations reduce the practical applicability of current artificial intelligence solutions in precision agriculture.

Motivated by these challenges, this study seeks to answer the following research questions:

**RQ1:** How can the integration of maize leaf images and environmental variables improve disease detection performance under real-world agricultural conditions?
**RQ2:** How does multimodal feature fusion influence maize disease classification performance compared with image-based and weather-based models?
**RQ3:** How can multimodal disease classification be integrated with an intelligent recommendation engine to generate context-aware, actionable agricultural recommendations for farmers?

To address these research questions, this study proposes a multimodal context-aware AI recommendation framework that integrates maize leaf images with environmental data collected from real agricultural fields in Morogoro, Tanzania. Unlike conventional disease classification systems that focus solely on diagnosis, the proposed framework extends the decision-making pipeline by automatically generating reliable and actionable recommendations for farmers. Furthermore, the generated recommendations are quantitatively evaluated to assess their safety, technical accuracy, relevance, actionability, and clarity. By integrating multimodal disease diagnosis, context-aware recommendation generation, and recommendation quality evaluation, the proposed framework bridges the gap between automated disease detection and practical agricultural decision support.

## Related work

Recent advancements in deep learning have significantly improved plant disease detection through convolutional neural networks (CNNs), transfer learning, and lightweight architectures [21–25]. CNN-based models such as MobileNetV2, ResNet, and EfficientNet have demonstrated high accuracy in classifying crop diseases by automatically learning discriminative visual features, including leaf texture, color variations, and lesion patterns from crop images [26–28]. Recent studies have further enhanced these architectures by incorporating explainable artificial intelligence techniques. For example, Ranaweera and Meedeniya [17] proposed a CNN-based framework for corn Gray Leaf Spot classification that integrates Gradient-weighted Class Activation Mapping (Grad-CAM) to improve model interpretability by highlighting the image regions influencing classification decisions. While this approach improves transparency and user confidence in AI-assisted diagnosis, it was evaluated using controlled datasets and remains focused on disease classification without integrating environmental context or providing post-classification decision support. Consequently, despite significant advances in image-based disease detection, challenges remain in developing models that generalize effectively under real-world agricultural conditions and support practical decision-making for farmers.

In addition to image-based methods, environmental data has been explored for disease prediction. Machine learning models using variables such as temperature, humidity, and rainfall can identify general patterns associated with disease occurrence [29], showed that environmental variables can be used to model crop disease trends. While these approaches contribute valuable contextual insights, their predictive performance is often limited due to overlapping environmental conditions across disease classes [30]. Similar to image-based models, most weather-based approaches also stop at prediction and lack mechanisms for translating results into practical agricultural guidance. Furthermore, weather-based models alone cannot capture the visual symptoms of plant diseases and therefore provide limited support for accurate diagnosis under diverse field conditions.

To overcome the limitations of single-modality systems, recent research has focused on multimodal learning, which integrates image and environmental data to improve classification performance. Studies show that combining multiple data sources enhances accuracy and robustness by leveraging complementary features [31,32]. Some emerging works also explore integrating deep learning with language-based systems to improve interpretability and user interaction [33]. However, even in multimodal frameworks, the primary emphasis remains on improving classification accuracy, with limited attention given to post-classification decision support. A few studies have attempted to develop agricultural decision-support systems [34,35]. These systems typically rely on predefined rule-based knowledge and are often implemented separately from predictive models. As a result, recommendations are not dynamically generated from multimodal prediction outputs, limiting their ability to provide personalized, context-aware guidance that adapts to specific disease conditions and environmental contexts.

## Methodology

### Dataset description

This study utilizes a combination of image and environmental data. The image dataset was sourced from the YEESI Lab Dataset [36], comprising 873 maize leaf images classified into three categories: Maize healthy leaves (661 images), maize streak virus–infected leaves (135 images), and Maize leaf aphid plants (77 images) obtained from Morogoro, as shown in Fig 1 and Table 1. The environmental dataset was obtained from the NASA POWER database [37] using the geographic coordinates of Morogoro, Tanzania, and consists of daily observations of temperature, relative humidity, rainfall, and solar radiation for the period August–September 2022 (61 days), as shown in Table 2. The environmental records were temporally aligned with the image acquisition period so that each image was associated with the corresponding daily weather conditions. Unlike many studies that rely on artificially balanced benchmark datasets, the original class distribution was preserved because it reflects the natural occurrence of healthy and diseased maize plants under real agricultural conditions. This enables the proposed framework to be evaluated in a realistic deployment scenario, providing a more representative assessment of its robustness and generalization capability for precision agriculture.

**Table 1:** Distribution of maize leaf image dataset showing the number of samples per disease category used in this study.

Data Type	Category	Number of Samples
Image Data	Healthy maize plants	661
	Maize streak virus–infected	135
	Aphid-infested maize plants	77
	Total Images	873

**Table 2:** First five records of the environmental dataset showing daily temperature, relative humidity, rainfall, and solar radiation.

date	temperature	humidity	rainfall	solar
2022-08-01	21.18	76.94	0.54	107.34
2022-08-02	21.31	70.16	0.07	205.15
2022-08-03	21.16	69.92	0.09	192.57
2022-08-04	20.78	74.73	0.01	156.06
2022-08-05	20.76	69.98	0.00	168.16

**Fig 1:**
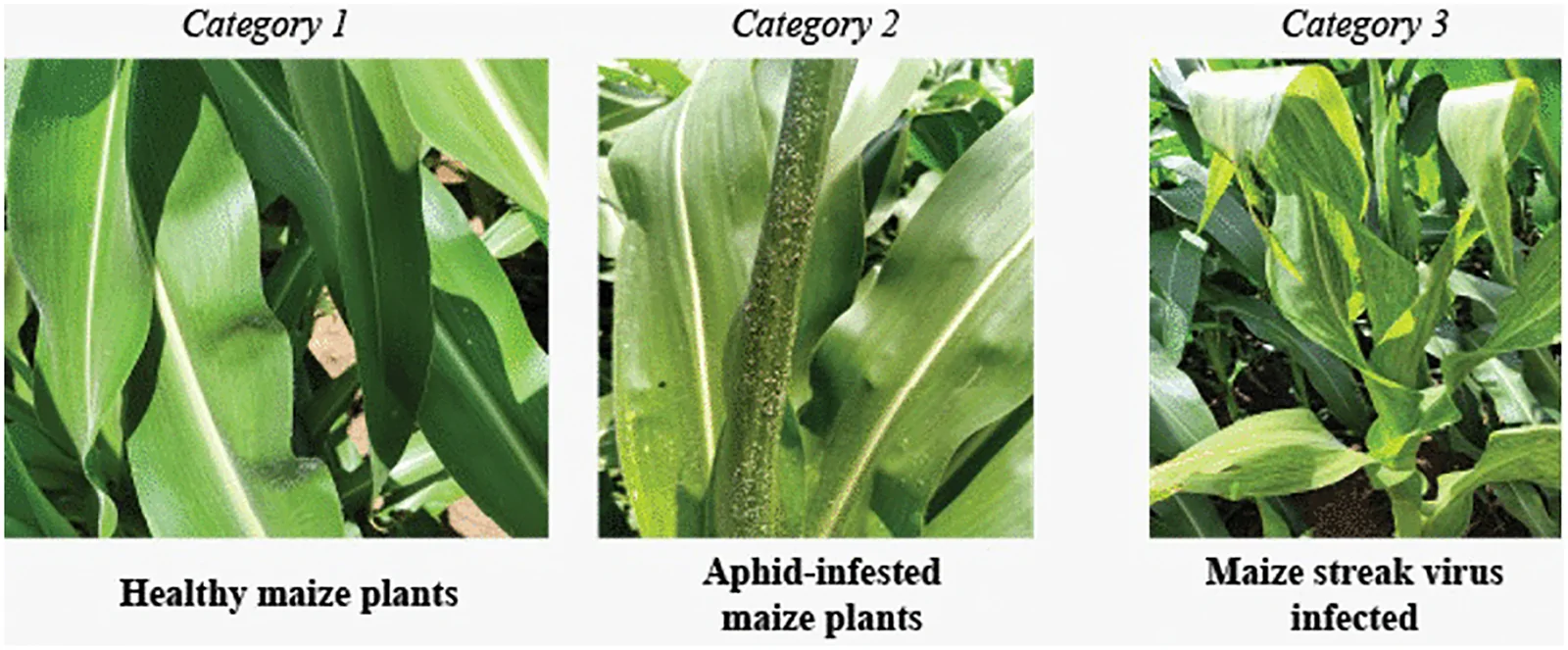
Sample maize leaf images used in the study: (left) a healthy leaf, (center) an aphid-infested leaf, and (right) a maize streak virus-infected leaf. We obtained the images from publicly available datasets hosted on Zenodo: *Maize Healthy* (https://zenodo.org/records/7893938/files/maize_healthy.zip?download=1), *Maize Leaf Aphid* (https://zenodo.org/records/7893938/files/maize_leaf_aphid.zip?download=1), and *Maize Streak Virus* (https://zenodo.org/records/7893938/files/maize_streak_virus.zip?download=1).

The average temperature was 22.62 °C, ranging from 20.45 °C to 25.18 °C, while relative humidity had a mean of 67.62% with values between 59.49% and 76.94%. Rainfall exhibited higher variability, with a mean of 0.74 mm/day and a maximum of 8.38 mm/day, indicating occasional precipitation events. Solar radiation averaged 200.51 W/m^2^, with values ranging from 107.34 to 286.55 W/m^2^. The standard deviations indicate moderate variability across most features, as shown in Table 3.

**Table 3:** Summary statistics of environmental variables used in the study, including temperature, humidity, rainfall, and solar radiation.

	Temp	Humidity	Rainfall	Solar
Count	61	61	61	61
Mean	22.62	67.62	0.74	200.51
Min	20.45	59.49	0	107.34
Max	25.18	76.94	8.38	286.55
Std	1.21	4.20	1.45	37.51

These statistics highlight relatively stable environmental conditions with overlapping ranges across disease classes, which explains the limited discriminative performance of the weather-based model and supports the need for multimodal integration.

The dataset was divided into training and testing sets using a stratified 80:20 split, where 80% of the data was used for model training and 20% for testing. The same data partition was maintained across all models to ensure a fair and unbiased comparison under identical experimental conditions while preserving the original class distribution. This evaluation protocol provides sufficient data for model learning while maintaining an independent test set for consistent performance assessment.

### Proposed system

The proposed system integrates image and environmental features to detect maize diseases using crop images and weather data. Given an input maize leaf image (I), the trained MobileNetV2 model extracts a 128-dimensional visual representation from its penultimate layer:


$$\begin{array}{c} \text{v}={\text{f}}_{\text{CNN}}(\text{I}),\;\;\text{v}\in R^{\text{128}} \end{array}$$

where $\left(f_{\text{CNN}}\right)$ represents the trained MobileNetV2 feature extractor and (𝐯) denotes the extracted visual feature vector. The environmental modality consists of four variables: temperature, relative humidity, rainfall, and solar radiation. Before fusion, each environmental variable is standardized using the training-set mean and standard deviation:


$$\begin{array}{c} {\text{w}}^{\prime }=\frac{{\text{w}}_{\text{j}}-\mu_{\text{j}}}{\sigma_{\text{j}}} \end{array}$$

where $\left({\text{w}}_{\text{j}}\right)$ is the original environmental value, $\left(\mu_{\text{j}}\right)$ is the training-set mean, and $\left(\sigma_{\text{j}}\right)$ is the training-set standard deviation. The fitted scaling parameters are subsequently applied to the test data to prevent information leakage.

The standardized environmental vector is then concatenated with the 128-dimensional image representation to form a 132-dimensional multimodal feature vector:


$$\begin{array}{c} \text{z}=\left[\text{v};{\text{w}}^{\prime }\right]\in R^{\text{1}\text{32}} \end{array}$$

The fused representation is then passed through two fully connected layers containing 128 and 64 neurons, respectively, with ReLU activation. The resulting representation is passed to a final softmax layer that produces the probability of each of the three maize conditions: healthy, aphid infestation, and maize streak virus infection. The class with the highest predicted probability is selected as the final disease classification. Based on the predicted disease and associated environmental conditions, a context-aware recommendation engine combines rule-based agronomic knowledge with a large language model to generate actionable natural-language recommendations for farmers. The recommendation generation process can be represented as:


$$\begin{array}{c} \text{R}=\text{G}\left(\text{D},{\text{w}}^{\prime },\text{K}\right) \end{array}$$

where (D) is the predicted disease condition, $\left({\text{w}}^{\prime }\right)$ represents the environmental context, (K) denotes the rule-based agronomic knowledge base, and (G) represents the recommendation generation process.

The generated recommendations are subsequently evaluated using a blind LLM-based evaluation framework across five dimensions: Safety, Technical Accuracy, Relevance, Actionability, and Clarity. The framework therefore provides an end-to-end decision-support pipeline integrating multimodal disease diagnosis, context-aware recommendation generation, and recommendation quality evaluation. Fig 2 illustrates the proposed system architecture.

**Fig 2:**
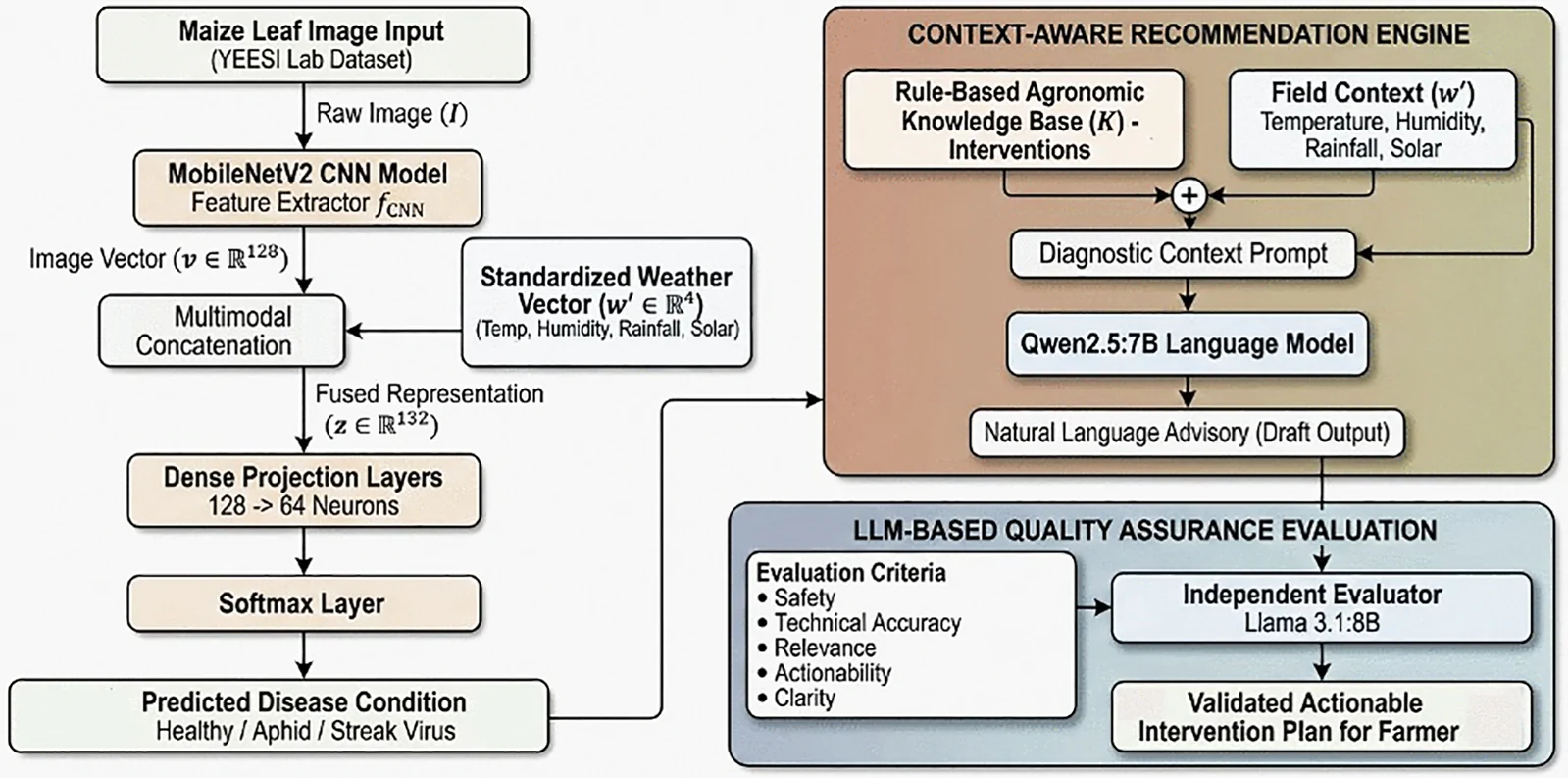
Proposed system architecture integrating image and environmental features for multimodal maize disease detection and context-aware recommendation generation.

### Weather-base model

Prior to developing the weather-based model, exploratory data analysis was conducted to examine the distribution of environmental variables across different disease classes. Fig 3 presents the distributions of temperature, relative humidity, rainfall, and solar radiation.

**Fig 3:**
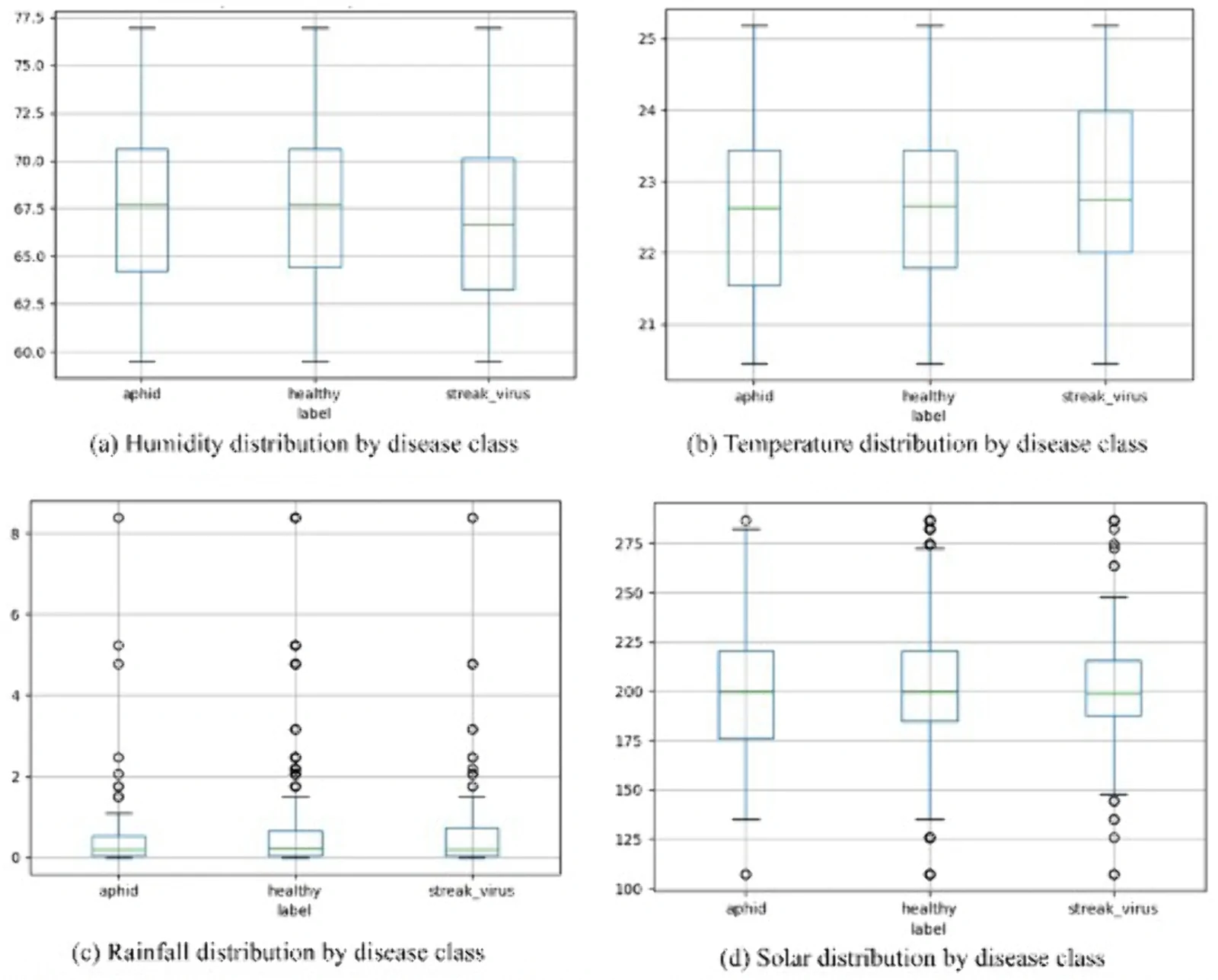
Boxplot distributions of environmental variables (humidity, temperature, rainfall, and solar radiation) across maize disease classes, showing substantial overlap among classes.

The overlap in the distributions of these variables among the classes, indicate that environmental factors alone have limited discriminative power for disease classification. A Random Forest classifier was used to predict disease classes based solely on environmental variables. The model was trained using the same train-test split used for other models to ensure methodological consistency. This model served exclusively as a baseline for comparison and was not incorporated into the multimodal fusion framework. The results suggests that while weather data contributes useful contextual information, it is insufficient as a standalone predictor and reinforces the need for a multimodal approach

### Image-based model

The image-based model utilizes a convolutional neural network built on MobileNetV2 architecture with transfer learning from ImageNet. During training, the model learns discriminative visual features such as leaf texture, color variations, and disease patterns to classify maize leaves into healthy, aphid-infested, or maize streak virus categories. Model training was monitored using accuracy and loss curves (Fig 7). An increasing training and validation accuracy, alongside decreasing loss, indicates effective learning and good generalization. Minimal divergence between training and validation curves suggests that the model avoids overfitting. Additionally, the ROC curves (Fig 9) demonstrate the model’s classification capability across different thresholds. The high AUC values (0.99–1.00) confirm that the model achieves excellent separability between classes, making it reliable for disease detection tasks.

### Multimodal fusion model

The multimodal fusion model (Fig 4) combines visual features extracted from the CNN with environmental variables to improve disease classification. Image features were extracted from the penultimate representation of the trained MobileNetV2-based image model, producing a compact 128-dimensional embedding for each maize leaf image. The environmental modality consisted of four numerical variables (temperature, relative humidity, rainfall, and solar radiation). Before multimodal fusion, these variables were standardized using the StandardScaler, where the scaler was fitted on the training data and subsequently applied to the testing data to ensure consistent feature scaling while preventing information leakage. The standardized weather vector was concatenated with the 128-dimensional image embedding to form a 132-dimensional multimodal feature vector. This fused representation was then processed through fully connected dense layers with 128 and 64 neurons, which acted as learnable projection layers to jointly optimize the contribution of both modalities during classification.

**Fig 4:**
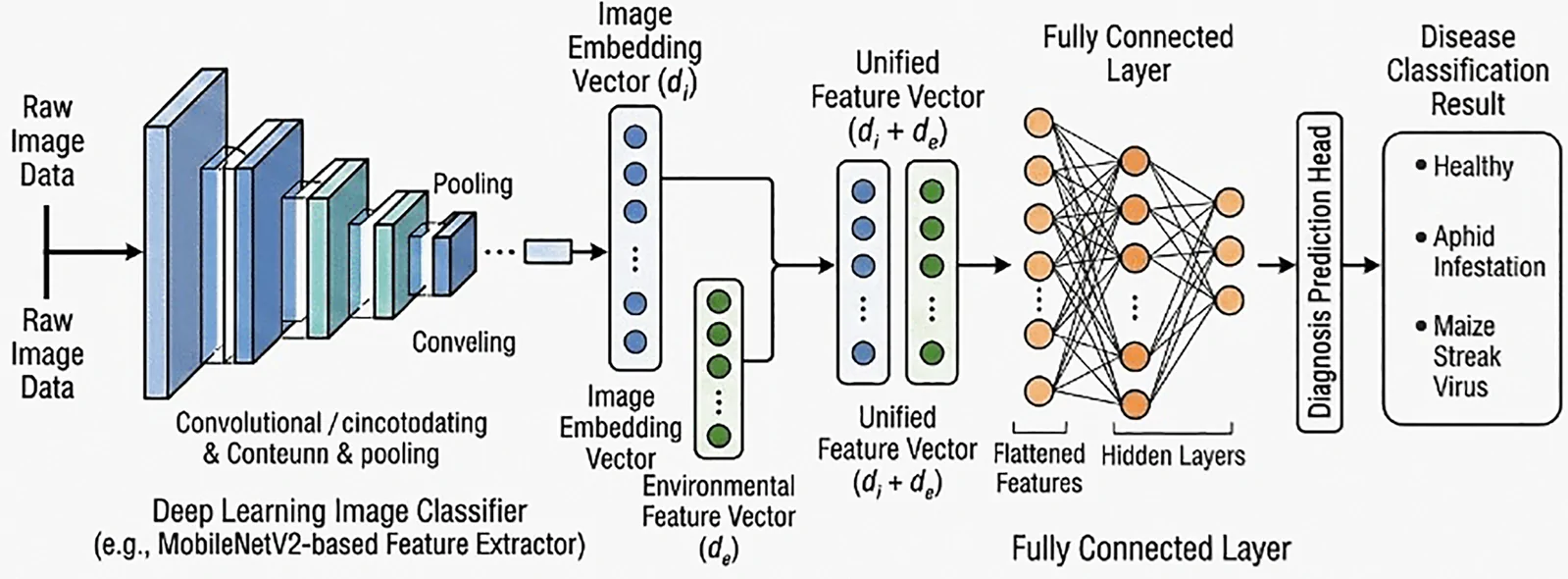
Multimodal fusion architecture integrating image features extracted and environmental variables, combined through a fusion layer and fully connected network for maize disease classification.

This fusion approach enables the model to jointly leverage visual disease characteristics and environmental context, enhancing overall predictive performance compared to using either modality independently.

### Recommendation engine

The recommendation engine (Fig 5) converts the predicted disease condition and associated environmental context into actionable agricultural guidance using a hybrid approach that combines rule-based agronomic knowledge with large language model-based natural language generation. For each predicted condition, predefined agronomic rules determine appropriate interventions, such as continued monitoring for healthy crops, pest management for aphid infestation, and disease management for maize streak virus.

**Fig 5:**
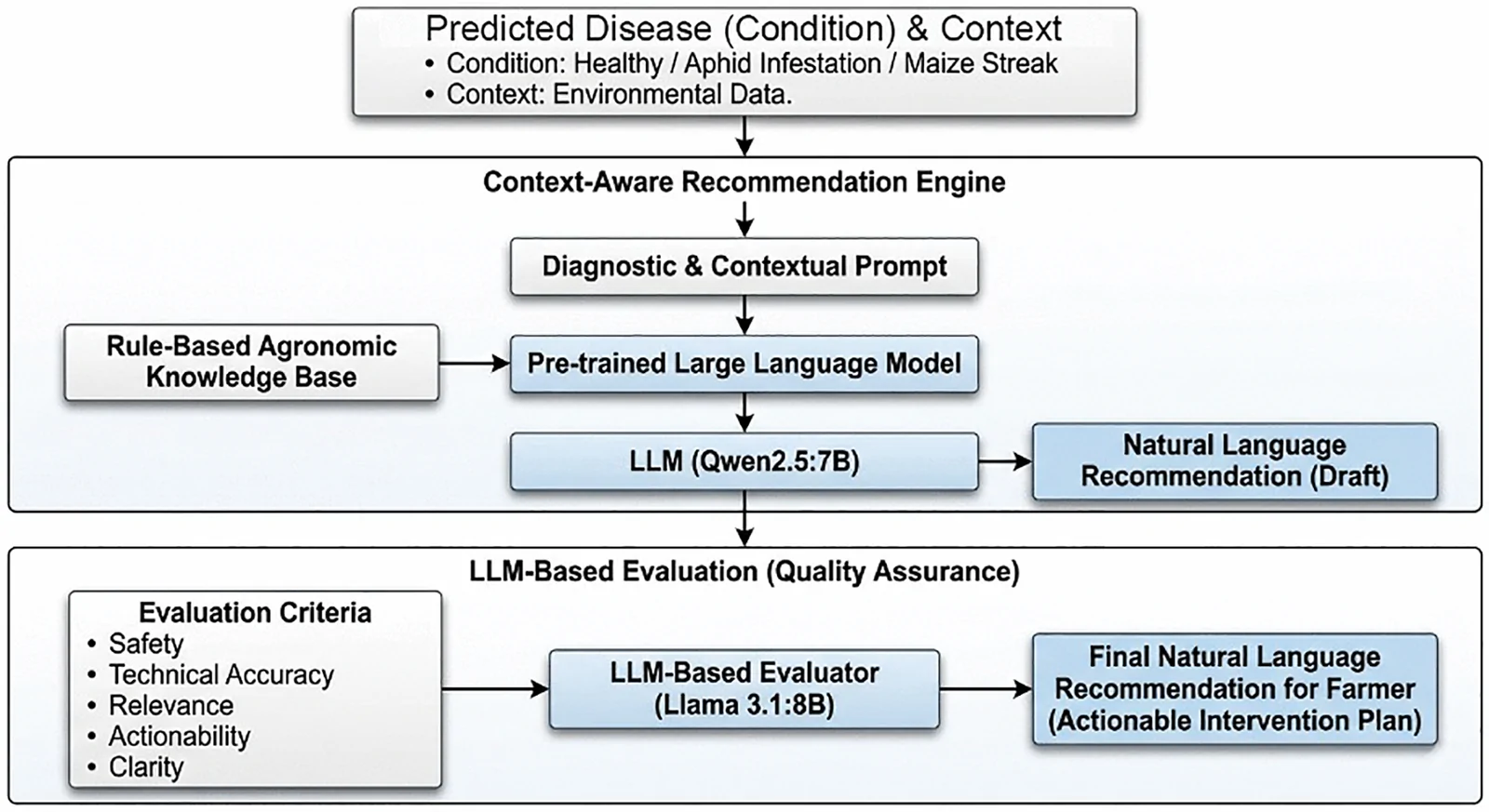
Recommendation engine workflow showing the transformation of predicted disease conditions into structured agronomic actions, followed by prompt construction, Qwen2.5:7B-based generation, and delivery of farmer-oriented recommendations.

To improve usability, Qwen2.5:7B is used to transform the structured agronomic guidance into natural-language recommendations. The model receives the disease condition, relevant environmental context, and rule-derived guidance as structured prompt information and produces a farmer-oriented advisory response. The rule-based component maintains consistency with predefined agronomic knowledge, while the language model improves readability and contextual presentation. The generated recommendations are subsequently evaluated independently using Llama 3.1:8B through a blind evaluation framework described in the following section.

## Results

### Weather-based model performance

The weather-based model achieved an overall accuracy of approximately 74% using temperature, relative humidity, rainfall, and solar radiation as input features in the Random Forest classifier. The model showed a strong bias toward the healthy class, with most healthy samples correctly classified, while aphid infestation and maize streak virus samples were frequently misclassified.

Feature importance analysis (Fig 6(b)) showed that humidity was the most influential variable, followed by temperature and solar radiation, while rainfall contributed the least to classification. The confusion matrix (Fig 6(a)) further showed that many diseased samples were incorrectly classified as healthy. These results indicate that, although environmental variables provide useful contextual information, they have limited discriminative capability when used alone for reliable maize disease detection.

**Fig 6:**
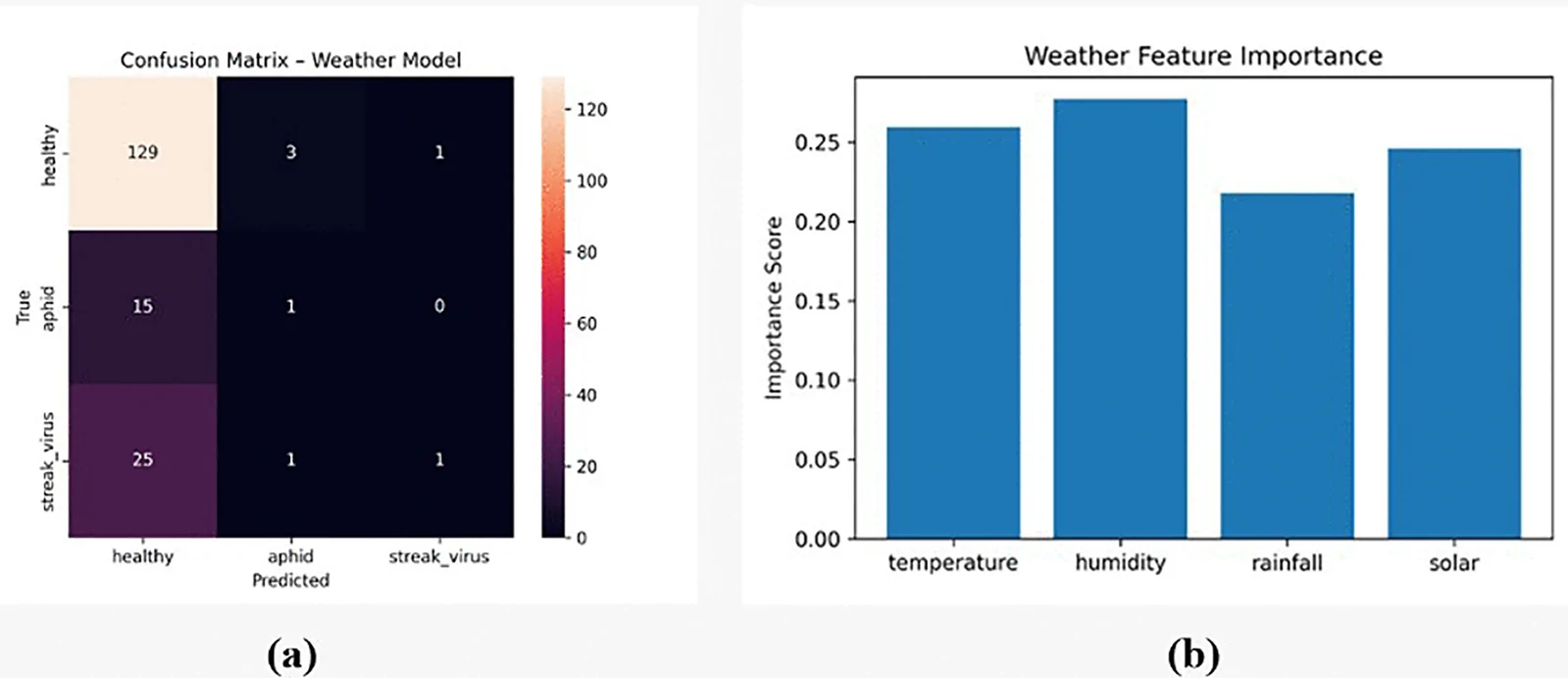
Weather-based Model Performance and Feature Importance. (a) confusion matrix and (b) feature importance of the environmental variables.

### Image-based model performance

The image-based model achieved strong classification performance in identifying maize crop conditions from leaf images. Using the MobileNetV2 architecture with transfer learning, the model achieved approximately 91% classification accuracy across the three classes: healthy maize plants, aphid infestation, and maize streak virus infection. The training and validation curves (Fig 7) demonstrated stable convergence, with validation performance closely following training performance, indicating minimal overfitting and good generalization capability.

**Fig 7:**
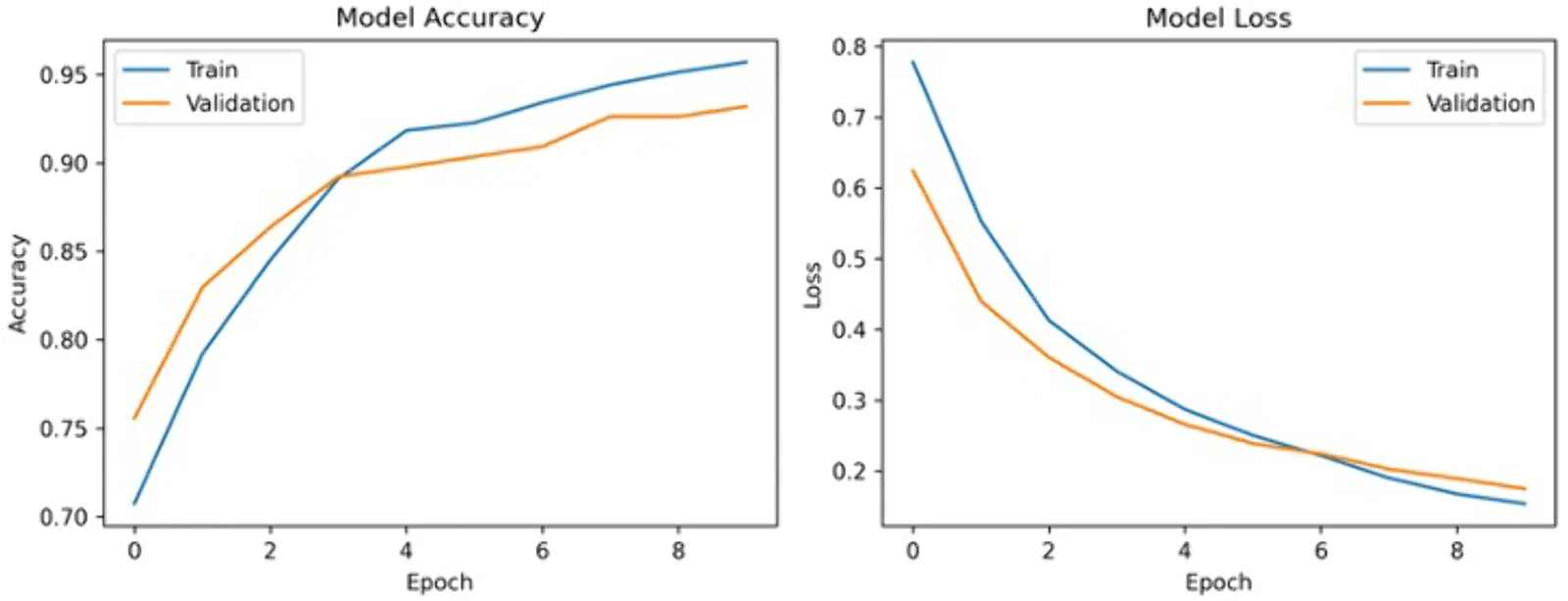
Training and validation accuracy and loss curves for the image-based model, showing steady performance improvement and good generalization across epochs.

The ROC analysis (Fig 8(a)) further confirmed the robustness of the model, with Area Under Curve (AUC) values approaching 1.0 across all classes. Healthy maize and maize streak virus categories achieved particularly high separability, while aphid-infested samples showed slightly lower discrimination due to visual similarities during early infestation stages.

**Fig 8:**
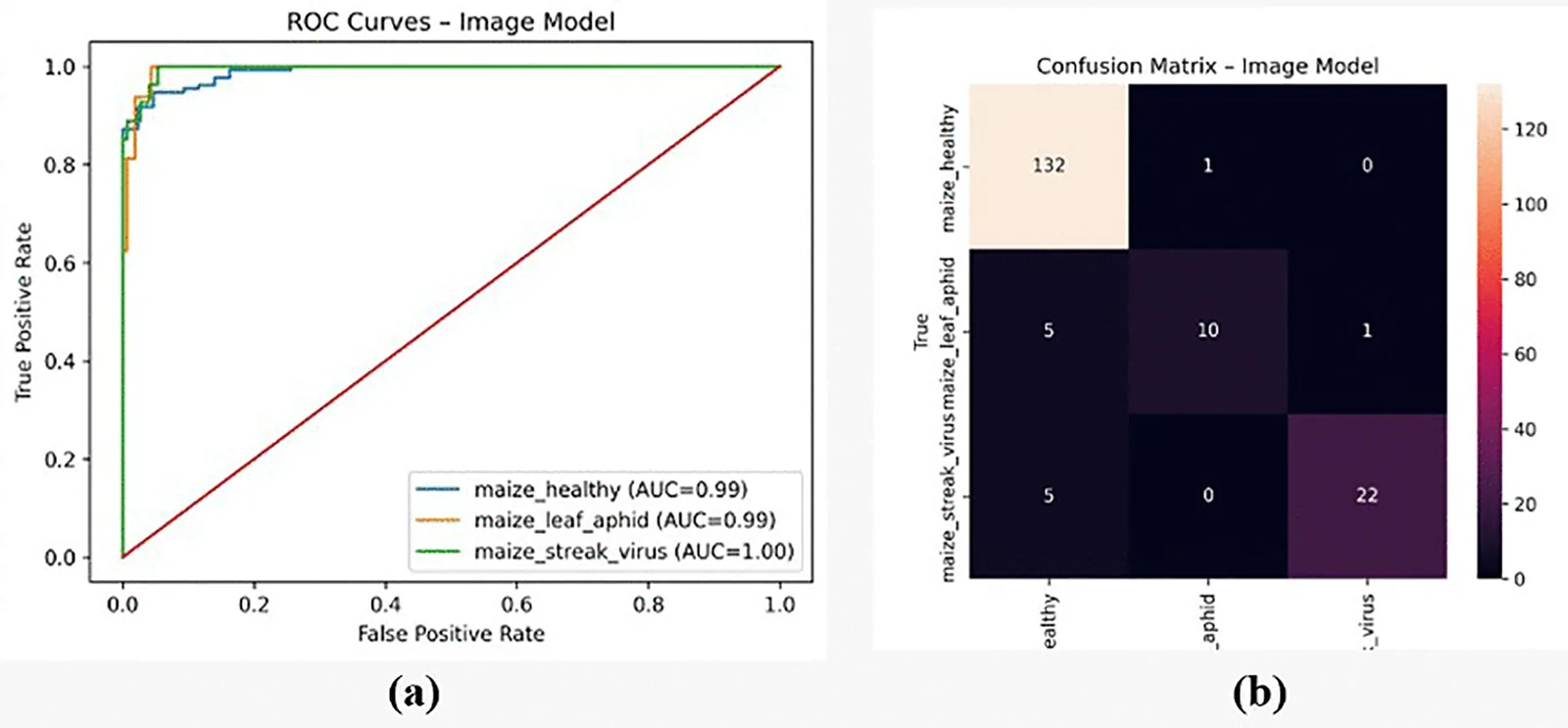
Image-Based Model Performance. (a) ROC curves showing high class discrimination with AUC values of 0.99–1.00, and (b) confusion matrix showing classification performance across the three maize conditions.

The confusion matrix (Fig 8(b)) analysis revealed that most healthy maize samples were correctly classified with very few false positives. Maize streak virus cases were also accurately detected, while aphid-infested leaves experienced a moderate level of misclassification into healthy and virus-infected categories. These findings demonstrate that visual deep learning models can effectively capture disease-related leaf characteristics such as discoloration, streak patterns, and texture variations.

### Multimodal fusion model performance

The multimodal fusion model (Fig 9) achieved the best overall performance, with approximately 94% classification accuracy. By integrating visual image features with environmental variables, the system significantly improved prediction reliability across all disease categories.

**Fig 9:**
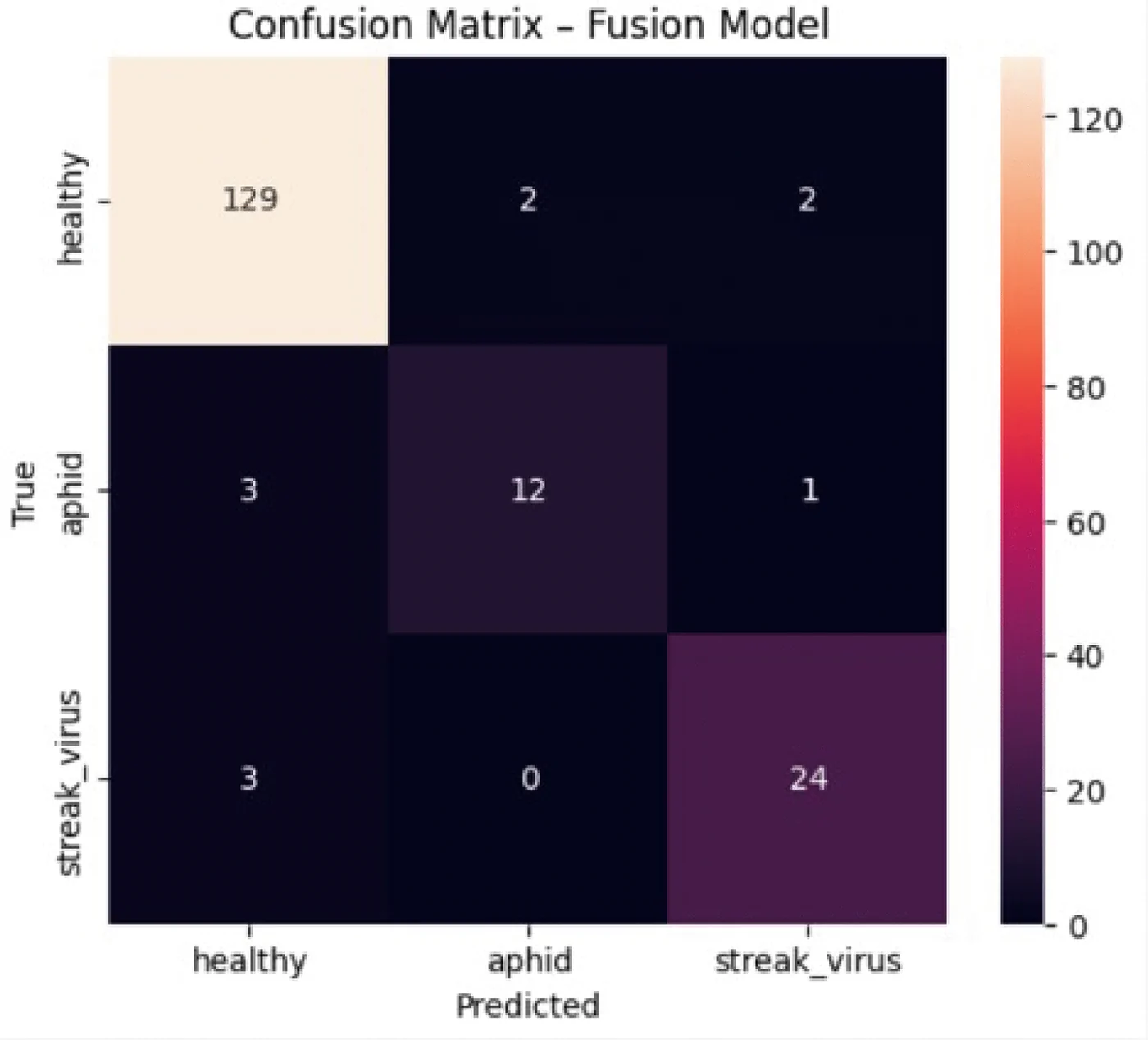
Confusion matrix of the multimodal fusion model showing improved classification accuracy and balanced performance across maize disease categories.

The fusion model reduced the class imbalance bias observed in the weather-based model and improved the classification of aphid-infested maize plants. Healthy maize samples maintained high classification accuracy, while maize streak virus predictions also improved with fewer false classifications. The integration of environmental context helped refine predictions in cases where image-based visual symptoms were ambiguous. Compared to the individual models, the multimodal system demonstrated stronger robustness, improved generalization, and more balanced predictive capability across disease classes.

### Effect of data augmentation and class-weighted learning

Additional experiments were conducted using data augmentation, class-weighted optimization, and a combination of both techniques to assess their effect on the naturally imbalanced maize dataset. Contrary to expectations, these techniques did not improve the performance of the proposed framework. Data augmentation reduced the image-based model accuracy from approximately 90% to 85%, while class-weighted optimization reduced accuracy to approximately 70%. The combined approach produced similar behavior. These results indicate that, for this dataset, additional imbalance-mitigation strategies introduced greater classification variability without improving overall performance. Therefore, the original model configuration was retained because it provided the best overall performance under the experimental conditions.

### Recommendation engine evaluation

The recommendation engine converts predicted disease conditions and environmental context into actionable agricultural recommendations using a hybrid approach that combines rule-based agronomic knowledge with Qwen2.5:7B for natural-language generation. The rule-based component provides the underlying agronomic guidance, while the language model converts the structured guidance into farmer-oriented advisory text without changing its intended meaning.

To quantitatively evaluate recommendation quality, 30 representative cases were randomly selected from the test dataset, covering healthy maize, aphid infestation, and maize streak virus under varying environmental conditions. Each generated recommendation was independently evaluated using Llama 3.1:8B in a blind evaluation setting. The evaluator received only the predicted disease condition, environmental variables, and generated recommendation, without access to the underlying agronomic rules. This design reduced the possibility of evaluating the recommendations based on knowledge of their generation process.

Recommendation quality was assessed using five criteria: Safety, Technical Accuracy, Relevance, Actionability, and Clarity as shown in Table 4**.** Each criterion was scored on a five-point scale, where 1 represented very poor performance and 5 represented excellent performance.

**Table 4:** Quantitative evaluation of generated recommendations.

Metric	Mean	Std
Safety	5.00	0.00
Technical Accuracy	4.37	0.49
Relevance	4.97	0.18
Actionability	4.67	0.48
Clarity	5.00	0.00

The recommendations achieved an overall mean quality score of 4.80/5.00 across the five evaluation dimensions. Safety and Clarity achieved the maximum mean score of 5.00, while Relevance achieved 4.97, indicating that the recommendations were consistently aligned with the predicted crop condition and environmental context. Actionability achieved a mean score of 4.67, demonstrating that the recommendations generally provided practical guidance. Technical Accuracy obtained a mean score of 4.37, representing the lowest of the five dimensions while remaining high overall and indicating opportunities for further refinement of the agronomic detail.

The high Safety and Technical Accuracy scores provide evidence that the rule-grounded recommendation approach can constrain unsupported agricultural advice while allowing the language model to produce more accessible natural-language responses. However, the blind LLM evaluation should be interpreted as a quality assessment rather than a formal guarantee of hallucination-free recommendations. The use of predefined agronomic rules provides an additional grounding mechanism that reduces the risk of unsupported recommendations, while independent evaluation provides quantitative evidence of their consistency, relevance, and practical usefulness.

### Model comparison

Table 5 presents the performance comparison of the weather-based, image-based, and multimodal fusion models using overall accuracy together with class-specific Precision, Recall, and F1-score. Since the maize dataset is naturally imbalanced, these additional metrics provide a more reliable assessment of model performance than overall accuracy alone. The weather-based model achieved an overall accuracy of 74%; Although the model successfully recognized healthy plants (F1 = 0.85), it achieved substantially lower performance for maize leaf aphid (F1 = 0.10) and maize streak virus (F1 = 0.07), indicating that environmental variables alone are insufficient for reliable disease classification.

**Table 5:** Performance comparison of weather-based, image-based, and multimodal fusion models using class-specific evaluation metrics.

Class	Precision	Recall	F1-score
**(a) Weather-Based Model**			
Healthy	0.76	0.97	0.85
Aphid	0.20	0.06	0.10
Maize Streak Virus	0.50	0.04	0.07
**Accuracy**			**0.74**
**(b) Image-Based Model**			
Healthy	0.89	0.99	0.94
Aphid	1.00	0.44	0.61
Maize Streak Virus	0.95	0.74	0.83
**Accuracy**			**0.91**
**(c) Multimodal Fusion Model**			
Healthy	0.96	0.97	0.96
Aphid	0.86	0.75	0.80
Maize Streak Virus	0.89	0.89	0.89
**Accuracy**			**0.94**

The image-based model significantly improved disease recognition, achieving an overall accuracy of 90%. It obtained F1-scores of 0.94 for healthy maize, 0.61 for maize leaf aphid, and 0.83 for maize streak virus, demonstrating the effectiveness of deep visual feature extraction. However, the relatively lower recall for the aphid class (0.44) suggests that early-stage aphid symptoms remain challenging to identify using image information alone.

The proposed multimodal fusion model achieved the best overall performance, with an accuracy of 94% and consistently strong class-specific metrics. The model achieved F1-scores of 0.96 for healthy maize, 0.80 for maize leaf aphid, and 0.89 for maize streak virus. Compared with the image-based model, multimodal fusion improved the F1-score of the minority aphid class from 0.61 to 0.80 and the maize streak virus class from 0.83 to 0.89, demonstrating that integrating environmental context with image features enhances disease discrimination and produces a more balanced classifier suitable for real-world agricultural deployment.

The results confirm that while image data provides strong predictive capability and environmental data offers contextual support, their combination leads to more robust and reliable disease classification. This demonstrates the effectiveness of multimodal learning in enhancing agricultural decision-support systems.

### Comparison with recent state-of-the-art studies

To position the proposed framework relative to recent advances, a comparison with representative studies published in 2026 was conducted. The selected studies represent the major research directions in intelligent crop disease diagnosis, including image-text multimodal learning, environmental sensor fusion, retrieval-augmented reasoning, and knowledge-guided decision support. The comparison highlights differences in data modalities, methodological design, recommendation capability, and classification performance.

As shown in Table 6, recent studies have primarily focused on improving disease classification through multimodal learning, environmental sensing, or knowledge-guided reasoning. Although these approaches demonstrate strong diagnostic performance, most terminate at disease recognition and do not provide actionable support for farmers. In contrast, the proposed framework combines multimodal disease classification with a rule-based recommendation engine that incorporates environmental conditions to generate context-aware management recommendations. Furthermore, recommendation quality is quantitatively validated through an independent blind LLM-based evaluation, providing objective evidence of recommendation safety, technical accuracy, relevance, actionability, and clarity. This integration of multimodal diagnosis, intelligent recommendation generation, and quantitative recommendation evaluation distinguishes the proposed framework as a comprehensive decision-support system for precision agriculture.

**Table 6:** Comparison of the proposed framework with recent state-of-the-art multimodal plant disease diagnosis studies.

Study (Year)	Method Used	Dataset Used	Modality	Recommendation Support	Classification Performance	Main Contribution
Tang et al. (2026) [38]	mIT-CMCA (DenseNet121 + DistilBERT + CSHA)	PlantVillage Maize (MPVD) + Maize Leaf-Field Dataset (MLFD)	Image + Text	✗	Accuracy = **93.67%**	Lightweight image-text multimodal framework for maize disease classification.
Berrocal et al. (2026) [39]	Enhanced YOLOv8n + VGG16 + Fuzzy Logic	Real maize field dataset	Image + Soil Moisture	✗	F1-score = **94.1%**	Multimodal disease severity assessment using RGB images and soil moisture sensing.
Guerrero-Ibañez et al. (2026) [40]	CNN + RNN + DCGAN	Public maize images + Environmental IoT data	Image + Temperature + Humidity + Soil Moisture	✗	Accuracy > 98.0%*	Edge-aware multimodal disease detection using environmental time-series and synthetic augmentation.
Chan et al. (2026) [41]	Structured Captioning + Retrieval-Augmented Multimodal Transformer	37,586 images (48 disease classes)	Image + Structured Text + Retrieval Knowledge	Partial	F1-score = **97.67%**	Retrieval-augmented multimodal framework for robust plant disease diagnosis.
Proposed Framework	MobileNetV2 + Early Weather Fusion + Recommendation Engine + Blind LLM Evaluation	Real maize field dataset (images + synchronized weather data)	Image + Weather	✓	Accuracy = 94.00%	Integrates multimodal disease classification with context-aware recommendation generation and quantitative evaluation of AI-generated agricultural recommendations.

## Discussion

This study was designed to address three research questions concerning multimodal disease detection and intelligent agricultural decision support. The findings demonstrate that integrating maize leaf images with environmental information improves disease classification while enabling the generation of practical, context-aware recommendations for farmers.

The results demonstrate that integrating visual and environmental information improves disease detection under real-world agricultural conditions. While the image-based model achieved strong classification performance (91%), incorporating environmental variables increased the overall accuracy to 94%. This improvement indicates that weather information provides complementary contextual information that helps distinguish disease conditions when visual symptoms alone are insufficient. Using field-collected data from Morogoro, Tanzania further demonstrates the applicability of the proposed approach under practical agricultural conditions rather than relying exclusively on curated benchmark datasets.

The multimodal fusion model consistently outperformed both the image-based and weather-based models. Statistical analysis further demonstrated that the observed improvement over the image-based model was statistically significant (McNemar’s test, *p* < 0.05), while the 95% confidence intervals confirmed the stability of the reported classification performance. These findings support the hypothesis that combining complementary modalities provides more reliable disease classification than relying on a single information source.

The proposed recommendation engine successfully transformed disease predictions into actionable agricultural recommendations by combining rule-based agronomic knowledge with Qwen2.5:7B for natural-language generation. Unlike conventional disease classification systems that terminate at prediction, the proposed framework extends the decision-making pipeline by producing farmer-oriented recommendations immediately after disease identification. The generated recommendations were subsequently evaluated using a blind Llama 3.1:8B evaluation framework, achieving an overall quality score of 4.80/5.00 across Safety, Technical Accuracy, Relevance, Actionability, and Clarity. These results indicate that the hybrid architecture produces recommendations that remain consistent with predefined agronomic knowledge while improving readability and usability.

The findings are consistent with previous studies demonstrating the effectiveness of deep learning for plant disease detection using leaf images [3,6,9]. Similar to Mayo et al. [6] and Mohanty et al. [9], the MobileNetV2-based image model effectively learned disease-related visual characteristics, including discoloration, streak formation, and texture variations. Likewise, the lower performance of the weather-based model agrees with previous studies [13,29,30], which reported that environmental variables alone provide useful contextual information but cannot reliably distinguish visually similar disease conditions because different diseases often occur under comparable climatic conditions.

The improved performance of the multimodal fusion model is also consistent with recent multimodal learning studies [17,21,32], which demonstrated that integrating complementary information sources improves classification robustness. However, unlike previous multimodal approaches that primarily focus on disease classification, the proposed framework integrates multimodal disease diagnosis, context-aware recommendation generation, and quantitative recommendation evaluation within a unified decision-support system. This distinguishes the proposed framework from existing studies by extending artificial intelligence beyond disease prediction toward practical agricultural decision support.

The additional experiments investigating data augmentation and class-weighted optimization further demonstrate that commonly adopted imbalance mitigation techniques are not universally beneficial. On the naturally imbalanced field dataset used in this study, both approaches reduced classification performance, indicating that preserving the original data distribution better reflected real-world deployment conditions.

Despite these promising findings, several limitations should be acknowledged. First, the study was conducted using a relatively small image dataset together with a 61-day environmental dataset collected from a single geographic region. Second, aphid infestation was represented by fewer samples than the other disease classes, potentially influencing classification performance. Third, although the recommendation engine demonstrated high quality under blind LLM-based evaluation, additional validation by agricultural experts and field deployment studies would provide stronger evidence of practical effectiveness. Future work will therefore focus on collecting multi-season and multi-location datasets, expanding disease coverage, incorporating additional environmental variables, and evaluating the framework through real-world farmer studies.

The findings demonstrate that integrating visual crop analysis, environmental context, and intelligent recommendation generation provides an effective end-to-end framework for agricultural decision support. Beyond improving disease classification accuracy, the proposed framework bridges the gap between automated diagnosis and practical agricultural advisory services, offering a promising foundation for future intelligent farming applications.

The proposed framework is intended for future deployment as a mobile application to support precision agriculture. Farmers will capture maize leaf images using a smartphone, while weather information will be retrieved automatically from weather services or entered manually when necessary. The application will perform multimodal disease classification and generate context-aware management recommendations, providing real-time decision support for maize disease management.

## Conclusion

This study proposed a multimodal context-aware AI recommendation framework for maize disease diagnosis that integrates maize leaf image analysis, environmental information, multimodal feature fusion, and intelligent recommendation generation within a unified decision-support system. The framework extends conventional disease classification by combining automated diagnosis with context-aware agricultural recommendations.

Experimental results demonstrated that integrating visual and environmental information improved disease classification compared with single-modality approaches. In addition, the hybrid recommendation engine successfully generated reliable, farmer-oriented recommendations, while blind LLM-based evaluation confirmed their high quality across safety, technical accuracy, relevance, actionability, and clarity. These findings demonstrate the potential of multimodal artificial intelligence to support practical agricultural decision-making under real-world conditions.

Agricultural institutions and extension services are encouraged to support the adoption of AI-assisted decision-support systems by promoting digital agriculture initiatives, strengthening agricultural data collection, and facilitating collaboration between researchers, agronomists, and extension officers. Such collaboration will enable continuous refinement of agronomic knowledge and improve the reliability of AI-generated recommendations.

Future work will focus on expanding the framework using larger multi-region and multi-season datasets, incorporating additional environmental variables, validating recommendations with agricultural experts and field trials, and deploying the framework as a mobile decision-support application for precision agriculture.
