# Supplementary material for: A multimodal context-aware AI recommender for smart farming
Source: PLoS One. 2026 Oct 8;21(10):e0359596. doi: 10.1371/journal.pone.0359596 (PMC13649118; doi:10.1371/journal.pone.0359596)
Supplement: Supplementary file 1 [file pone.0359596.s002.docx]

Reviewer Comments

**REVIEWER I**

| **SN** | **Reviewer Comment** | **Response** |
| --- | --- | --- |
| **1** | Some of the references in the reference list are missing bibliographic information (e.g., Ref. [3]). | We thank the reviewer for identifying this issue. The reference list has been carefully reviewed and corrected. Missing bibliographic information, including complete titles, journal names, publication years, volume/issue numbers, page or article numbers, and DOIs (where available), has been added to comply with the journal's reference formatting requirements. |
| **2** | Introduction – Refer more latest related work from 2026, 2025 and clearly state how your contribution covers the latest gaps. | We sincerely thank the reviewer for this valuable suggestion. The Introduction has been substantially revised to incorporate recent studies published in **2025 and 2026** covering deep learning, multimodal learning, explainable AI, and large language models for plant disease diagnosis. The revised literature review also identifies a clear gap in the latest research: although recent approaches increasingly integrate multiple data modalities, most continue to focus primarily on disease detection and classification, with limited integration of **context-aware recommendation generation and quantitative evaluation of AI-generated agricultural advice.** The revised Introduction explicitly positions our contribution within this gap by proposing a multimodal context-aware AI recommender framework that integrates image-based disease diagnosis, environmental information, multimodal feature fusion, rule-based agronomic knowledge, and LLM-assisted recommendation generation, followed by blind LLM-based evaluation of recommendation quality. These revisions clarify the novelty and relevance of the proposed framework in relation to the latest research. |
| **3** | In the introduction, state the research questions addressed in this study. In the discussion section, describe how you fulfilled the said research questions, using the proposed methodology and the obtained results. | We appreciate the reviewer's valuable suggestion. The Introduction has been revised to explicitly state the research questions that guided this study. In addition, the Discussion section has been expanded to explain how each research question was addressed through the proposed multimodal methodology and validated using the experimental results. These revisions improve the clarity of the study objectives and strengthen the connection between the methodology, results, and conclusions. |
| **4** | Related studies – it would be nice to discuss the techniques used in latest studies such as <https://doi.org/10.1109/SCSE70081.2026.11499862> | We thank the reviewer for this valuable suggestion. The Related Work section has been revised to include recent studies published in 2025–2026, including the suggested work. We now discuss the underlying techniques employed in these studies, such as CNN-based disease classification with Grad-CAM explainability, multimodal learning, and large language model integration. Furthermore, we contrast these approaches with our proposed framework by highlighting that, unlike existing studies, our work combines multimodal disease classification with a hybrid recommendation engine that generates context-aware, actionable recommendations for farmers. |
| **5** | The image dataset contains 661 healthy samples but only 135 for Maize Streak Virus and 77 for Aphids. Because the data is highly imbalanced, relying primarily on global Accuracy (~94%) can be highly misleading. Therefore, provide class-specific metrics (Precision, Recall, and F1-Score) for all three models in Table 3. | We thank the reviewer for this valuable observation. The manuscript has been revised by expanding Table 3 to include class-specific Precision, Recall, and F1-score for the weather-based, image-based, and multimodal fusion models. These additional metrics provide a more informative assessment of model performance, particularly for the minority disease classes.  It is important to note that the dataset was preserved in its original class distribution because it represents the natural occurrence of healthy and diseased maize plants under real agricultural field conditions. Rather than artificially balancing the dataset, which may overestimate real-world performance, our objective was to evaluate the proposed framework under realistic deployment scenarios.  The manuscript has been revised accordingly. |
| **6** | Why didn’t you use any data augmentation or class-weighted loss functions during training to protect the minority disease classes from being ignored by the optimizer. Is it possible to run the model again and get the results by addressing this. | We sincerely thank the reviewer for this valuable suggestion. Following the recommendation, we conducted additional experiments to investigate the effects of data augmentation, class-weighted loss, and their combination on the naturally imbalanced maize disease dataset. These experiments were performed using the same MobileNetV2 architecture, training configuration, and evaluation protocol to ensure a fair comparison.  The results indicated that while these imbalance mitigation techniques influenced class-specific performance, they did not improve the overall balance between classification accuracy and minority-class recognition for our real-world dataset. Specifically, data augmentation reduced the overall classification accuracy from 90% to 85%, while class-weighted training further reduced the accuracy to approximately 70% by introducing excessive false-positive predictions for the minority classes. The combination of augmentation and class-weighted loss exhibited similar behavior.  These findings suggest that the naturally imbalanced dataset reflects the actual distribution of maize diseases observed under field conditions in Morogoro, Tanzania. Consequently, aggressive imbalance mitigation strategies caused the model to overcompensate for minority classes, reducing its ability to correctly recognize the dominant healthy class and lowering the overall generalization performance.  Based on these comparative experiments, we retained the original image model because it provided the best trade-off between overall accuracy and class-specific performance. The manuscript has been revised to include class-specific Precision, Recall, and F1-score metrics together with an additional discussion of these experiments. |
| **7** | The environmental data spans only 61 days (August to September 2022). Can you state exactly how a single daily weather observation vector was paired with individual images? If multiple images were taken on the same day, did they share identical weather vectors? If yes, discuss the risk of data leakage or artificial correlation during the random 80:20 train/test split. | We thank the reviewer for this insightful observation. The environmental dataset consisted of daily weather observations obtained from the NASA POWER database for the Morogoro study area between August and September 2022. Each maize leaf image was associated with the weather measurements corresponding to its date of acquisition. Consequently, multiple images collected on the same day shared the same environmental feature vector while retaining their own image data and disease labels.  We acknowledge that this alignment may introduce correlation among samples collected on the same day. Because the image and environmental datasets were randomly divided into training and testing subsets using an 80:20 split, it is possible that images acquired on the same date appeared in both subsets and therefore shared identical environmental attributes. This could potentially introduce a degree of information leakage through the environmental modality.  However, the environmental variables constitute only one component of the proposed multimodal framework. The primary discriminatory information is derived from the maize leaf images, while the environmental variables provide complementary contextual information rather than unique identifiers of disease classes. This is supported by the independent evaluation of the weather-only model, which achieved substantially lower performance (74% accuracy; macro F1-score = 0.34) than both the image-based model and the multimodal fusion model. These results indicate that the environmental features alone have limited predictive capability and primarily serve to enhance, rather than dominate, the classification process.  To clarify this aspect, the manuscript has been revised by explicitly describing the image-weather alignment procedure and by acknowledging this potential limitation in the Discussion section. |
| **8** | Multimodal Fusion Specifics: In Section 3.6, you state that image embeddings from MobileNetV2 and weather features are concatenated. Generally, MobileNetV2 outputs high-dimensional embeddings, and the weather vector consists of just 4 variables (temperature, humidity, rainfall, solar radiation). Simple concatenation would allow the image features to completely overwhelm the weather features numerically. Specify if any feature scaling, dimensionality reduction, or projection layers were applied to the text/image vectors before concatenation. | We thank the reviewer for this important observation. In the proposed framework, the weather variables (temperature, relative humidity, rainfall, and solar radiation) were standardized using the StandardScaler, where the scaler was fitted on the training set and subsequently applied to the testing set to prevent information leakage.  Rather than directly concatenating the original high-dimensional MobileNetV2 features, the proposed framework extracts a compact 128-dimensional image embedding from the trained image model. This embedding is then concatenated with the four standardized weather variables, producing a 132-dimensional multimodal feature vector.  The fused representation is subsequently processed through fully connected dense layers (128 and 64 neurons), which serve as learnable projection layers that automatically optimize the relative contribution of both modalities during end-to-end training. Consequently, feature scaling together with the compact embedding representation prevents numerical dominance of the image modality and enables effective integration of visual and environmental information.  The manuscript has been revised to clarify these implementation details. |
| **9** | In this study, a Random Forest was used for the weather-only model, while deep neural layers were used for the fusion model. Can you justify this selection in technical terms. Why a simple MLP was not used for the weather-only model. Explain how the Random Forest outputs were seamlessly integrated if late fusion was considered instead of early concatenation. | We sincerely thank the reviewer for this important observation. We agree that the methodology description could have been clearer.  The Random Forest model was not integrated into the multimodal fusion network. Instead, it was developed as an independent baseline model to evaluate the predictive capability of environmental variables alone. The purpose of this model was to quantify the discriminative power of weather information without visual features and to provide a baseline for comparison with the image-based and multimodal approaches.  The proposed multimodal framework employs early feature-level fusion, rather than late decision-level fusion. Specifically, deep image embeddings extracted from the trained MobileNetV2 model were concatenated directly with the standardized environmental variables (temperature, relative humidity, rainfall, and solar radiation). The resulting multimodal feature vector was subsequently processed by fully connected neural network layers to perform disease classification. Consequently, the outputs of the Random Forest classifier were not used during multimodal fusion.  Regarding the selection of Random Forest for the environmental baseline, the environmental dataset consisted of only four numerical variables and a relatively limited number of observations. Random Forest was selected because of its robustness for structured tabular data, ability to capture nonlinear relationships, resistance to overfitting on small datasets, and interpretability through feature importance analysis. In contrast, multilayer perceptrons generally require substantially larger datasets to achieve stable generalization and would have introduced unnecessary model complexity for the weather-only baseline.  To avoid confusion, Section 3.6 has been revised to explicitly distinguish the independent weather baseline from the proposed multimodal fusion framework and to clarify that the fusion strategy is based on early feature concatenation rather than late fusion. |
| **10** | Please provide evaluation metrics for LLM-generated recommendations in Section 4.4, showing numerical validation of the text output quality. | Thank you for this valuable suggestion. We have extended Section 4.5 by introducing a quantitative evaluation of the generated recommendations. Thirty representative recommendation cases covering all disease classes were evaluated using an independent Large Language Model (Llama 3.1) under a blind assessment protocol.  The evaluator received only the predicted disease, environmental conditions, and generated recommendation without access to the underlying agronomic rules. Recommendation quality was assessed using five criteria: Safety, Technical Accuracy, Relevance, Actionability, and Clarity on a five-point Likert scale.  The resulting quantitative evaluation has been incorporated into the revised manuscript as Table 4 and discussed in Section 4.5. |
| **11** | Is it possible to include human expert evaluation (e.g., scoring by Agronomists on a 1–5 scale for Safety, Technical Accuracy, and Actionability) or automated language metrics (like BERTScore or G-Eval) to guarantee the system does not produce agricultural hallucinations? | Thank you for the insightful recommendation. Human expert evaluation by agronomists would indeed provide the strongest external validation. However, access to multiple certified agronomists was beyond the scope of the present study. Instead, following recent literature on LLM evaluation, we introduced an automated blind evaluation protocol using Llama 3.1 as an independent evaluator.  The evaluator scored recommendation quality across Safety, Technical Accuracy, Relevance, Actionability, and Clarity without access to the original agronomic rules. This evaluation provides quantitative evidence that the generated recommendations preserve agronomic correctness while maintaining high readability. We acknowledge that future work will incorporate human expert evaluation by agricultural specialists to further validate recommendation quality under real farming conditions. |
| **12** | Include a comparison table to compare the proposed results with the existing latest studies. | Thank you for this valuable suggestion. We have addressed this comment by adding a new comparison table (**Table 6)** in Section 4.6, comparing the proposed framework with four recent state-of-the-art studies published in 2026. The comparison includes the methodology, dataset, data modality, recommendation capability, classification performance, and key contribution of each study. Unlike previous approaches that primarily focus on disease classification or diagnostic reporting, the proposed framework integrates multimodal disease classification with context-aware recommendation generation and quantitative evaluation of AI-generated agricultural recommendations.  A more comprehensive experimental comparison of the proposed approach against existing methods using standardized performance metrics is part of the **next objective of this research,** which focuses on evaluating the proposed model against existing approaches. The present study therefore provides a state-of-the-art comparison based on the performance metrics reported in the selected recent studies, while the broader comparative evaluation will be undertaken as part of the subsequent research objective. |
| **13** | **What is the possibility of deploying this model in real-world as in** [**https://doi.org/10.1109/SCSE70081.2026.11499862**](https://doi.org/10.1109/SCSE70081.2026.11499862)**?** | Thank you for the suggestion. We have clarified the real-world deployment potential of the proposed framework. The system is intended to be deployed as a mobile application where farmers can capture maize leaf images, retrieve weather information automatically or enter it manually, and receive real-time disease diagnosis together with context-aware management recommendations. This deployment perspective has been added as future work under discussion section in the revised manuscript |
| **14** | **Clearly specify the exact GPT model version utilized and state the core hyperparameter settings used during inference.** | Thank you for highlighting the importance of reproducibility. We have revised the manuscript to explicitly specify the large language model used for recommendation generation and evaluation. The revised manuscript states that Llama 3.1 (8B), deployed locally through Ollama, was used. This information has been added to the implementation details in the methodology. |
| **15** | **Proofread the paper for typos.** | Thank you for the suggestion. The manuscript has been carefully proofread and revised to correct typographical, grammatical, punctuation, and formatting errors. We also improved sentence clarity, standardized terminology, and ensured consistency in figures, tables, and references throughout the manuscript. |
| **16** | **It would be better to provide the data availability link for the 61-day environmental dataset CSV file to an open repository (such as Zenodo alongside the YEESI dataset link).** | Thank you for the valuable suggestion. The dataset used in this study are publicly available at <https://doi.org/10.5281/zenodo.7729284> and the weather dataset and codes are available at <https://github.com/mhagama/research> |

**REVEWER II**

| **SN** | **Reviewer Comment** | **Response** |
| --- | --- | --- |
| **1** | An accuracy of approximately 91% is reported for the image-based model and approximately 94% for the multimodal model. However, it has not been demonstrated whether this performance difference is statistically significant. Confidence intervals, p-values, or appropriate statistical comparisons have not been provided. | We appreciate the reviewer's valuable observation. The reported improvement from the image-based model (91%) to the proposed multimodal model (94%) demonstrates a consistent performance gain across all evaluation metrics, including precision, recall, and F1-score. We acknowledge that the original manuscript did not include statistical significance testing or confidence interval analysis.  This has now been explicitly recognized as a limitation of the current study in the revised manuscript. Future work will include formal statistical validation, such as confidence interval estimation and hypothesis testing, using larger multi-site datasets to further quantify the statistical significance of the observed performance improvements. |
| **2** | The evaluation was conducted using only a single 80%/20% training-test split, and neither k-fold cross-validation nor an independent validation dataset was used. | We thank the reviewer for this valuable comment. A stratified 80%/20% train-test split was deliberately adopted to ensure a fair comparison by evaluating the image-based, weather-based, and multimodal models on the same data partition while preserving class distribution. Since the primary objective was to assess the contribution of multimodal fusion under identical experimental conditions, this evaluation protocol was considered appropriate. We acknowledge that k-fold cross-validation and independent external validation would further strengthen the generalizability analysis and have identified these as directions for future work. |
| **3** | The dataset used in the study is relatively small and unbalanced across classes. In particular, the number of samples in the aphid class is quite low. In addition, the meteorological data covers only 61 days of observations and a single geographic region. This limits the model’s generalizability to different regions and different growing conditions. It is recommended that these limitations be discussed in greater detail and, if possible, supported by additional validation experiments. | We thank the reviewer for this important observation. We acknowledge that the dataset is relatively small, exhibits class imbalance (particularly for the aphid class), and that the environmental observations cover only a 61-day period from a single geographical region.  These characteristics reflect the natural field conditions under which the data were collected and were intentionally preserved to evaluate the proposed framework under realistic deployment scenarios rather than artificially balanced conditions. To address this concern, the Discussion section has been expanded to explicitly describe these limitations, their potential impact on model generalizability, and future plans to validate the framework using larger, balanced, multi-season, and multi-location datasets. |
| **4** | The technical details of the multimodal fusion model should be explained more comprehensively. In particular, the feature dimensions used, the fusion method, hyperparameters, learning rate, batch size, number of epochs, data augmentation strategies, and the training process should be provided in detail. This information is necessary for the reproducibility of the study. | We thank the reviewer for this valuable suggestion. The methodology has been substantially revised to improve the reproducibility of the proposed framework. The revised manuscript now provides detailed information on the feature dimensions, multimodal fusion strategy, image embedding extraction, weather feature preprocessing, network architecture, hyperparameter settings (learning rate, batch size, number of epochs, optimizer), training procedure, and data augmentation techniques. These additions provide a comprehensive description of the multimodal fusion model and facilitate reproducibility of the proposed approach. |
| **5** | Although the recommendation engine was presented as one of the study’s significant contributions, it has not been sufficiently evaluated. The version of the GPT model used, the prompt design, and how the accuracy of the recommendations was verified were not explained. An evaluation by agricultural experts or a user-centered performance analysis would significantly strengthen the study’s scientific contribution. | We thank the reviewer for this valuable comment. The recommendation engine has been substantially expanded in the revised manuscript. A dedicated evaluation section has been added describing the recommendation generation process, the LLM version and inference settings, prompt design, and a quantitative blind evaluation framework based on Safety, Technical Accuracy, Relevance, Actionability, and Clarity. The evaluation demonstrates that the generated recommendations are context-aware, agronomically consistent, and grounded in the rule-based recommendation engine. We agree that evaluation by professional agronomists and user-centered field studies would further strengthen the practical validation of the recommendation system and have identified these as important directions for future work. |
| **6** | It is also recommended that the study include comparisons with stronger baseline methods and present an ablation study demonstrating the contribution of each component of the proposed architecture. These analyses would more convincingly demonstrate the extent to which the multimodal approach actually contributes. | We thank the reviewer for this valuable suggestion. The revised manuscript has been strengthened by including additional comparisons with recent state-of-the-art studies published in 2026. Furthermore, the experimental evaluation includes separate image-based, weather-based, and multimodal models, all trained and evaluated under identical experimental conditions. These comparative experiments demonstrate the contribution of each modality and the performance improvement achieved through multimodal feature fusion. We acknowledge that a more comprehensive component-wise ablation study, including systematic evaluation of individual environmental variables and alternative fusion strategies, would provide further insight into the contribution of each component.  A more comprehensive experimental comparison of the proposed approach against existing methods using standardized performance metrics is part of the next objective of this research, which focuses on evaluating the proposed model against existing approaches. The present study therefore provides a state-of-the-art comparison based on the performance metrics reported in selected recent studies, while the broader comparative and component-wise evaluation will be undertaken as part of the subsequent research objective. |
| **7** | There are also shortcomings regarding data accessibility. Although access to the image dataset was provided, the meteorological dataset, the final data structure used for multimodal fusion, training codes, model weights, and the prompts for the recommendation system were not shared. Therefore, it does not appear possible to fully reproduce the study. | Thank you for the valuable suggestion. The dataset used in this study are publicly available at <https://doi.org/10.5281/zenodo.7729284> and the weather dataset and codes are available at <https://github.com/mhagama/research> |
| **8** | Finally, the English text of the article should undergo careful editing. There are numerous grammatical and terminological errors throughout the text (e.g., “Fusion model” instead of "Fusion modal" "Weather-Based Model" instead of "Weather-Base Modal," and "plant pasts" instead of "plant pests"). These corrections will improve the article’s readability and academic quality. | Thank you for the suggestion. The manuscript has been carefully proofread and revised to correct typographical, grammatical, punctuation, and formatting errors. We also improved sentence clarity, standardized terminology, and ensured consistency in figures, tables, and references throughout the manuscript. |
